# Management of transient bone osteoporosis: a systematic review

**DOI:** 10.1093/bmb/ldad012

**Published:** 2023-06-16

**Authors:** Filippo Migliorini, Gianluca Vecchio, Christian David Weber, Daniel Kämmer, Andreas Bell, Nicola Maffulli

**Affiliations:** Department of Orthopaedic, Trauma, and Reconstructive Surgery, RWTH University Hospital, 52074 Aachen, Germany; Department of Orthopaedics and Trauma Surgery, Academic Hospital of Bolzano (SABES-ASDAA), 39100 Bolzano, Italy; Department of Medicine, Surgery and Dentistry, University of Salerno, 84081 Baronissi, SA, Italy; Department of Orthopaedic, Trauma, and Reconstructive Surgery, RWTH University Hospital, 52074 Aachen, Germany; Department of Orthopaedic and Trauma Surgery, Eifelklinik St.Brigida, 52152 Simmerath, Germany; Department of Orthopaedic and Trauma Surgery, Eifelklinik St.Brigida, 52152 Simmerath, Germany; Department of Medicine, Surgery and Dentistry, University of Salerno, 84081 Baronissi, SA, Italy; School of Pharmacy and Bioengineering, Keele University Faculty of Medicine, ST4 7QB Stoke on Trent, England; Barts and the London School of Medicine and Dentistry, Centre for Sports and Exercise Medicine, Mile End Hospital, Queen Mar y University of London, 275 Bancroft Road, London E1 4DG, UK

**Keywords:** transient, osteoporosis, syndrome, bisphosphonates

## Abstract

**Introduction:**

Transient bone osteoporosis (TBO) is characterized by persistent pain, loss of function, no history of trauma and magnetic resonance image (MRI) findings of bone marrow edema.

**Source of data:**

PubMed, Google scholar, EMABSE and Web of Science were accessed in February 2023. No time constrains were used for the search.

**Areas of agreement:**

TBO is rare and misunderstood, typically affecting women during the third trimester of pregnancy or middle-aged men, leading to functional disability for 4–8 weeks followed by self-resolution of the symptoms.

**Areas of controversy:**

Given the limited evidence in the current literature, consensus on optimal management is lacking.

**Growing points:**

This systematic review investigates current management of TBO.

**Areas timely for developing research:**

A conservative approach leads to the resolution of symptoms and MRI findings at midterm follow-up. Administration of bisphosphonates might alleviate pain and accelerate both clinical and imaging recovery.

## Introduction

Transient bone osteoporosis (TBO) is a rare, misdiagnosed, self-limiting condition of unclear etiology. TBO is characterized by pain,^1^ loss of function, absence of previous trauma,^2^ osteopenia on plain radiography and bone marrow edema at magnetic resonance imaging (MRI).^3^ TBO typically affects middle-aged men^4^ or, less commonly, women during the third trimester of pregnancy and the immediate post-partum period.^5^^,^^6^ TBO usually presents with sudden-onset pain in weight-bearing areas, especially in the lower limb, often radiating distally.^7^^,^^8^ In most patients, TBO leads to functional disability within 4–8 weeks, followed by a gradual disappearance of the symptoms in the following 6–12 months.^9^^,^^10^ The clinical examination might demonstrate limited effusion. Imaging, including plain radiographs, bone scans and MRI, is used for the diagnosis. MRI is fundamental for the diagnosis, evidencing nonspecific and localized bone marrow edema hyperintense in T2 sequences.^6^^,^^7^^,^^11^^,^^12^ TBO should be differentiated from bone osteonecrosis and metastases.^13^^,^^14^ Other less common conditions to consider for the differential diagnosis are regional migratory osteoporosis, reflex sympathetic dystrophy, arthritis of various etiologies such as septic arthritis, osteomyelitis and insufficiency fracture.^15^ Nevertheless, it remains a diagnosis of exclusion, usually delayed, partly from the lack of awareness.^16^ Despite a benign prognosis, the long clinical course causes prolonged disability. Given the limited evidence in the current literature, consensus on optimal management is lacking. In most cases, conservative management allows the resolution of symptoms within 6–12 months.^17^ The main conservative approaches include restricted weight-bearing, anti-resorptive medications and analgesics.^6^ This systematic review investigates current management of TBO.

## Methods

### Eligibility criteria

All the clinical studies, which investigated modalities for the management of TBO, were accessed. According to the author's language capabilities, articles in English, German, Italian, French and Spanish were eligible. Levels I to IV of evidence studies, according to Oxford Centre of Evidence-Based Medicine,^18^ were considered. Reviews, opinions, letters, editorials, animals, *in vitro*, biomechanics, computational and cadaveric investigations were not considered.

### Search strategy

This systematic review was conducted according to the Preferred Reporting Items for Systematic Reviews and Meta-Analyses: the 2020 PRISMA statement.^19^ The following PICO algorithm was established for the databases search:

- P (Problem): TBO,
- I (Intervention): clinical management,
- C (Comparison): conservative modalities,
- O (Outcomes): clinical outcome.

In April 2023, PubMed, Web of Science, Google Scholar, Embase were accessed with no constrains. The following keywords were used in combination using the Boolean operators AND/OR: transient osteoporosis syndrome, transient bone edema syndrome, management, treatment, weight bearing, surgery, conservative, outcome, pharmacology, drugs.

### Selection and data collection

Two authors (F.M. & G.V.) independently conducted the databases search. All the resulting titles were screened by hand and the abstract of the articles, which matched the topic was read. The full texts of the abstracts of interest were accessed. The bibliographies of the full-text articles were also screened by hand. Any disagreements were discussed and settled by a third senior author (N.M.).

### Data items

Two authors (F.M. & G.V.) independently performed data extraction. The following data were extracted: author and year, name of the journal and study design, length of the follow-up, number of included patients, type and number of joints, mean age and body mass index (BMI) of the included patients, number of women, type of treatment and main findings.

### Study risk of bias assessment

The methodological index for non-randomized studies (MINORS) was performed to evaluate the quality of the included article.^20^ The MINORS involves eight items for non-comparative studies and 12 items for comparative studies. The MINORS optimal global score is 16 points for the non-comparative studies and 24 points for the comparative studies.

### Synthesis methods

For descriptive statistics, the IBM software version 25 was used. The arithmetic mean and standard deviation were used for continuous variables.

## Results

### Study selection

**Fig. 1:**
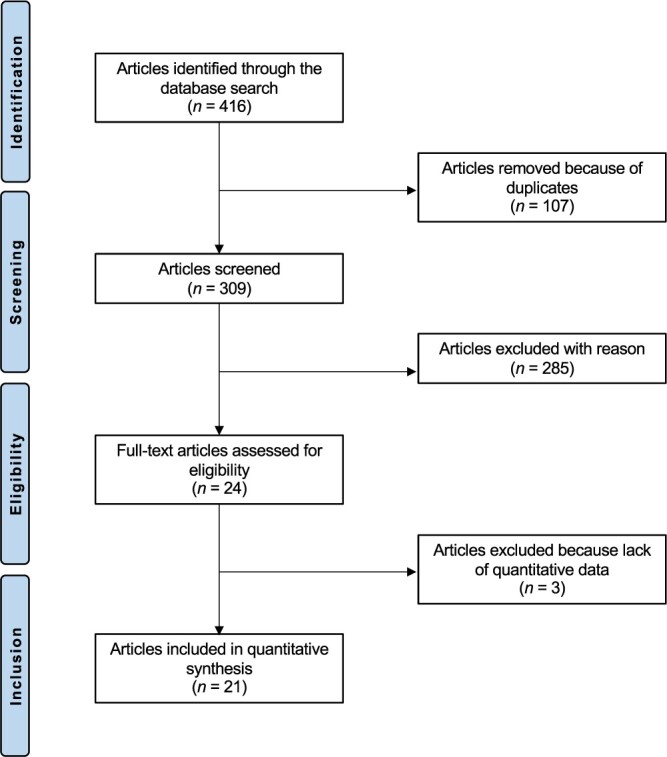
Flow chart of the literature search.

The literature search resulted in 416 clinical studies. Duplicate records (*N* = 107) were excluded. A further 285 articles were excluded with reason: not matching the topic (*N* = 181), inappropriate study design (*N* = 98), language limitation (*N* = 2) and full-text not available (*N* = 1). A further three articles did not report quantitative data under the outcome of interest, and were thus excluded. This left 21 articles for inclusion. The flow chart of the literature search is shown in Figure 1.

### Methodological quality assessment

Based on the MINORS scale, the 20 non-comparative studies had a medium score of 6.6 and the only comparative study scored 20 points. The MINORS attested to the present study a low quality of the methodological assessment (Table 1).

**Table 1:** Methodological quality assessment (0 = not reported; 1 = reported but inadequate; 2 = reported and adequate)

Author et al., year	1	2	3	4	5	6	7	8	9	10	11	12	Total
Agarwala et Vijayvargiya et al. (2019)^30^	2	2	2	2	0	2	2	2					14
Al-Dourbi et al. 2021^33^	2	0	0	2	0	2	0	0					6
Altan et al. (2019)^34^	2	0	0	2	0	2	0	0					6
Bashaireh et al. (2020)^28^	2	2	2	2	0	2	2	1	2	2	2	1	20
Berman et al (2016)^3^	2	2	0	2	0	2	0	0					8
Bin Abdulhak et al. (2011)^1^	2	0	0	2	0	2	0	0					6
Ergin et al (2010)^38^	2	0	0	2	0	2	0	0					6
Hong et al. (2019)^23^	2	0	0	2	0	2	0	0					6
McWalter et Hassan (2009)^22^	2	0	0	2	0	2	0	0					6
Okada et al. (2016)^25^	2	0	0	2	0	2	0	0					6
Paiva et al. (2020) ^26^	1	0	0	2	0	2	0	0					5
Pallavi et al. (2012)^16^	2	0	0	2	0	2	0	0					6
Pande et al. (2017)^31^	2	0	0	2	0	2	0	0					6
Paoletta et al. (2020)^32^	2	0	0	2	0	2	0	0					6
Reddy et al (2015)^21^	1	0	0	2	0	2	0	0					5
Seok et al. (2011)^9^	2	0	0	2	0	2	0	0					6
Thomas et Ninan (2019)^39^	1	0	0	2	0	2	0	0					5
Vaishya et al. (2017)^27^	1	2	2	2	0	2	2	0					11
Van Wagenen et al. (2013)^24^	2	0	0	2	0	2	0	0					6
Willis-Owen et al. (2008)^7^	2	0	0	2	0	2	0	0					6
Wright et al. (2021)^34^	2	0	0	2	0	2	0	0					6

### Synthesis of results

Data from 65 patients (74 treated joints) were collected. About, 23% (15 of 65) were women. The mean length of the follow-up was 14.4 months. The mean age of the patients was 38.1 ± 10.4 years, and the mean BMI was 28.8 kg/m^2^. The conservative management of TBO proved to be effective at middle and long-term follow-up, evaluating the resolution of symptoms and MRI findings. Treatment with bisphosphonates seems to alleviate pain and accelerate both clinical and imaging recovery. Generalities, patient characteristics and main results of the included studies are shown in Table 2.

**Table 2:** Generalities, patient characteristics and main results of the included studies

Author et al., year	Journal	Study design	Follow-up (months)	Treatment	Patients (*n*)	Mean age	Mean BMI	Female (*n*)	Main findings
Agarwala et Vijayvargiya et al. (2019)^30^	*Ann Rehabil Med*	Retrospective	35	Zoledronic acid, Vit D, calcium	19 patients, 19 hips	42.1		2	Intravenous single dose administration of zoledronic acid provides early pain relief and complete reversal of TBO. Consequently, zoledronic acid is proposed as a new paradigm in the management of TBO. At a mean follow-up of 35 months, all the patients were pain-free and were able to continue with their normal routine activities without any limitations.
Al-Dourbi et al. 2021^33^	*BMJ Case Rep*	Retrospective		Uncemented total hip arthroplasty	1 patient, 1 hip	24	40	1	Six weeks after surgery, the plain radiographs showed a well-implanted uncemented left THA. The osteopenia of the proximal femur and the acetabulum were almost resolved, while still femorotibial and fibular osteopenia are present
Altan et al. (2019)^34^	*J Orthop Case Rep*	Retrospective	24	Core decompression	1 patient, 1 hip	33		0	At 2 years follow-up, there was no hip pain and thrombocyte count was 463 103/μL. X-ray has no specific changes and MRI shows a decreasing of the intensity of edema
Bashaireh et al. (2020)^28^	*Open Access Rheumatol*	Retrospective		Protected weight bearing, NSAIDs (5 patients)	15 patients, 16 hips	41	28.2	1	Full recovery was 5.8 weeks for hip drilling or core decompression and was 48.3 weeks for three patients who received conservative treatment. Two of the five treated conservatively had not achieved full or near-full recovery.
				Core decompression (10 patients)					
Berman et al. (2016)^3^	*Bone Rep*	Retrospective	38	Risedronate, Vit D, calcium	1 patient, 1 hip	35		0	After 3 months, the patient reported complete resolution of his symptoms and was able to proceed with pain-free weight bearing, 3 years later bone marrow edema on contralateral side. Same management, same improvement of complains after 2 months
			12	Vit D, calcium	1 patient, 1 knee	64		0	spontaneous resolution of his knee pain over the next month
Bin Abdulhak et al. (2011)^1^	*Oman Med J*	Retrospective	18	Analgesics, rest, prophylactic antithrombotic therapy	1 patient, 2 hips	28		1	One month later, the pain regressed and we were able to stop the analgesics after 7 weeks, showed complete recovery of the left hip and a significant reduction in the right sided trochanteric edema
Ergin et al. (2010)^35^	*J Turk Ger Gynecol Assoc*	Retrospective	6	Alendronate, vit D, calcium, NSAIDs, calcitonin, bed rest, no weight bearing	1 patient 1 hip	34		1	symptoms and MRI findings regressed 6 months following delivery
Hong et al. (2019)^23^	*Arthroplast Today*	Retrospective	12	Protected weight bearing, physical therapy, NSAIDs	1 patient, 1 hip	40		1	At the 2-month follow-up, the patient reported no hip pain after strictly adhering to touchdown weight-bearing restrictions. More than 1 year from her initial presentation, the patient remains pain free with no complaints
McWalter et Hassan et al. (2009)^22^	*Ann Saudi Med*	Retrospective	12	Protected weight bearing, physical therapy, NSAIDs	1 patient, 1 hip	33		1	One year after her initial presentation her symptoms had completely resolved
Okada et al. (2016)^25^	*Case Rep Orthop*	Retrospective	12	Protected weight bearing and NSAIDs	1 patient, 2 hips	25	20.9	1	The left hip was fully improved with a normal range of motion three months after onset, and full weight bearing without pain was possible. By four months, the range of motion in bilateral hips had completely. Follow-up X-ray and magnetic resonance image were normal. Recovered
Paiva et al. (2020)^26^	*J Orthop Case Rep*	Retrospective	24	No weight-bearing, NSAID	1 patient, 1 hip	48		1	Twenty months after childbirth, she presented a considerable improvement of the complaints, with good mobility/function of the left hip joint, with no need for walking aid, and with a second MRI revealing reduction of intra-articular free fluid and downsizing of the extensive signal change that affected almost the entire left femoral head, which had a size of 1 × 2 cm (Fig. 3). A later follow-up revealed a complete resolution
Pallavi et al. (2012)^16^	J Obstet Gynaecol India.	Retrospective	6	Protected weight-bearing, gradual mobilization, NSAID	1 patient, 1 hip 2 vertebrae	30		1	At the 1-month follow-up, her ambulatory function had improved remarkably and a repeat CT pelvis showed focal osteopenic changes, but less than before. At the 6-month follow-up, she was asymptomatic and had resumed her routine activities
Pande et al. (2017)^31^	*Malays Orthop J*	Retrospective	7	Alendronate, no weight bearing, physical therapy, NSAID, sodium, calcium, vit D	1 patient, hip	43		0	At last follow-up, the patient had complete relief of pain with full painless range of movements. At further review five months after diagnosis and treatment the patient was mobilizing with full weight bearing. He had a full and painless range of movements at the right hip. Repeat MRI scan confirmed complete resolution of changes
Paoletta et al. (2020)^32^	*BMC Musculoskelet Discord*	Retrospective	2	Clodronate, calcium, Vit D, physical therapy, hormone replacement	1 patient, 1 hip	46	22.2	0	After 3 months of treatment, significant improvement of clinical and radiological outcomes was observed. We suggested for the first time a role of subclinical hypothyroidism as novel contributory factor for the onset of this condition.
Reddy et al. (2015)^21^	*BMJ Case Rep*	Retrospective	24	Protected weight bearing, physical therapy, NSAIDs	1 patient, 1 hip	38		1	The patient is completely painless and symptom free at 2-year follow-up
Seok et al. (2011)^9^	*Ann Rehabil Med*	Retrospective	6	Zoledronate, protected weight bearing, hot packs, shock wave therapy	1 patient, 1 hip	46		0	Normal gait was achieved one week after the treatments. No pain was observed upon weight bearing despite a slight pain in the inguinal area 2 weeks after treatments. Follow-up MRI of the hip joint taken after 6 months showed complete resolution.
Thomas et Ninan et al. (2019)^37^	Euro J Rheumatol.	Retrospective	2		1 patient, 1 hip	31		0	Patient was advised conservative treatment and his symptoms completely subsided within 4 weeks. Follow-up MRI performed 2 months later showed complete resolution of the marrow edema in the right femoral head and no residual subarticular bone changes were observed.
Vaishya et al. (2017)^27^	*Indian J Orthop*	Retrospective	30	Protected weight bearing, physical therapy, NSAIDs	12 patients, 14 hips	41		1	None progressed to femoral head collapse, arthritic changes, or avascular necrosis of the head. The average period for complete resolution of symptoms was 17.1 weeks. All the patients could join their work back after conservative management, with no recurrence reported in an average follow-up 1.3 years.
Van Wagenen et al. (2013)^24^	*J Can Chiropr Assoc*	Retrospective	4	Protected weight bearing, NSAIDs	1 patient, 1 hip	59		0	The patient reported a full resolution of his symptoms at the follow-up appointment after 4 months. Regular and moderate manual labor provoked no symptomatic flair or recurrence.
Willis-Owen et al. (2008)^7^	*Cases J*	Retrospective	6	Closed reduction and internal fixation	1 patient, 2 hips	34		1	Full weight bearing on the right, and partial weight bearing on the left was initiated on the first postoperative day, and maintained for the first 12 weeks. At six months, she was pain free with no evidence of avascular necrosis or implant failure.
Wright et al. (2021)^36^	*BMJ Case Rep*	Retrospective	6	Lower segment caesarean section and internal fixation	1 patient, 2 hips	24	32.8	1	The patient was reviewed in clinic after a further six months and reported excellent progression with full pain-free range of motion in both hips.

## Discussion

According to the main findings of the present study, conservative management leads to the resolution of symptoms and MRI findings at midterm follow-up. Administration of bisphosphonates might alleviate pain and accelerate clinical recovery and imaging appearance.

Several case reports referred to conservative management with limited weight bearing, physical therapy, and nonsteroidal anti-inflammatory drugs (NSAIDs), reporting full recovery in all patients at approximately one year follow-up.^16^^,^^21–26^^,^^39^Vaishya et al.^27^ conducted a study analyzing 12 hips in 14 patients with hip TBO treated conservatively. All the patients returned to work with a complete resolution of symptoms at 17.1 weeks.^27^ At 1.3-year follow-up, no recurrence was observed in any patient.^27^ Baishareh et al. performed an observational study on 15 patients with symptomatic hip TBO.^28^ The mean age of the patients was 41 years.^28^ Ten of 15 patients underwent core decompression and 5 patients were treated conservatively.^28^ The time needed for full recovery was 5.8 weeks for those who underwent drilling and 48.3 weeks for the three patients treated conservatively.^28^ Two patients who underwent conservative management did not achieve full recovery at the time of follow-up.^28^ The author hypothesized that hip core decompression could be considered as a treatment modality to achieve faster recovery in patients with hip TBO.^28^

Treatment with bisphosphonates has shown promising results, shortening the duration of symptoms.^8^^,^^9^^,^^29^ Agarwala et al.^30^ administered an intravenous single dose of zoledronic acid to 19 adults with hip TBO. At an average of 2.8 weeks, symptoms ceased, with no adverse events and recurrence to the last follow-up at a mean of 35 months. About, 84% (16 of 19) of patients did not demonstrate evidence of TBO at MRI. Berman et al.^3^ described the case of a 35-year-old male patient who presented with progressive and disabling pain in his left hip. Risedronate 35 mg once weekly for 12 weeks was administered. Calcium and vitamin D were also supplemented.^3^ After 3 months, the patient reported a complete resolution of symptoms and disability.^3^ Three years later, following onset of TBO contralaterally, the same treatment was administered, obtaining the same results at 2 months follow-up.^3^ Furthermore, they presented the case of a 64-year-old with a two-week history of progressively increasing left knee pain. A high-resolution MRI of his left distal femur revealed deterioration in bone microarchitecture (manifested by trabecular loss and disruption).^3^ The regional bone mineral density of his left lateral femoral condyle was 0.96 g/cm^2.^^3^ The patient continued with routine calcium and vitamin D supplementation.^3^ The patient reported spontaneous resolution of his knee pain over months.^3^ A further regional knee bone density of the left lateral femoral condyle showed marked improvement of 1.63 g/cm^2^ at one year of follow-up.^3^ Pande et al.^31^ reported a 43-year-old male patient with hip TBO managed by weight-bearing restriction, physiotherapy, administration of alendronate 10 mg daily, and calcium and vitamin D supplementations. At seven weeks, the patient evidenced a complete remission of symptoms with a complete recovery of the full range of motion.^31^ At five months follow-up, no evidence of TBO was observed at MRI and the administration of alendronate was discontinued.^31^ At seven months follow-up, the patient resumed normal activities. Paoletta et al.^32^ described a 46-year-old man with a diagnosis of hip TBO treated with intramuscular clodronate 200 mg for a month and weight-bearing restriction. Significant pain relief, improved motion, and a significant reduction of bone edema at MRI scans was observed at 2 months follow-up.^32^ Seok et al.^9^ presented the case of a 46-year-old male with a diagnosis of hip TBO, treated with a single dose of intravenous zoledronate 5 mg, weight-bearing restriction and hot packs.^9^ Despite a slight pain in the inguinal area, no pain was observed during weight bearing at the two-week follow-up. Additional 2 weeks of limited weight bearing were recommended.^9^ At four weeks follow-up, the pain in the inguinal area disappeared. At six months follow-up, no evidence of TBO was observed at MRI.^9^

TBO and especially transient osteoporosis of the hip frequently occurs in pregnant women in the third trimester or in the immediate postpartum period.^1^^,^^7^^,^^16^^,^^26^^,^^33^^,^^34^^,^^38^ Pregnancy limits the choices of pharmacotherapy. Brodell et al.^35^ suggested that the benefit of radiographic imaging may outweigh the potential risks in the third trimester of pregnancy. Furthermore, gold standard diagnostic imaging is via MRI, which should be considered a safe modality in the third trimester.^36^ Cesarean section is preferable to vaginal delivery to avoid the risk of trauma to the weak head of the femur in cases of TBO of the hip.^1^

The present study has several limitations. The overall quality of the evidence was low. Most of the available data come from case reports and retrospective studies. In this respect, results are not fully generalizable. Given the rarity of TBO, high-quality studies on a larger scale are arduous. Between studies, patient characteristics were heterogeneous. Given these limitations, the results of the present study must be considered with caution.

## Conclusion

A conservative approach leads to the resolution of symptoms and MRI findings of TBO at midterm follow-up. Administration of bisphosphonates seems to alleviate pain and accelerate both clinical and imaging recovery.

## CRediT for author contributions

Filippo Migliorini (Conceptualization, Data curation, Project administration, Supervision, Writing—review & editing), Gianluca Vecchio (Data curation, Formal analysis, Investigation), Christian Weber (Investigation, Methodology, Software), Daniel Kaemmer (Data curation, Investigation, Software, Visualization), Andreas Bell (Investigation, Methodology, Validation, Visualization), Nicola Maffulli (Conceptualization, Formal analysis, Writing—original draft).

## Conflict of interest statement

The authors declare that they have no conflict of interest.

## Funding

No external source of funding was used.

## Data availability

The data underlying this article are available in the article and in its online supplementary material.

## Ethical approval

This article does not contain any studies with human participants or animals performed by any of the authors.

## Informed consent

For this type of study, informed consent is not required.
